# RNA Polymerase III, Ageing and Longevity

**DOI:** 10.3389/fgene.2021.705122

**Published:** 2021-07-06

**Authors:** Yavuz Kulaberoglu, Yasir Malik, Gillian Borland, Colin Selman, Nazif Alic, Jennifer M. A. Tullet

**Affiliations:** ^1^Department of Genetics Evolution and Environment, Institute of Healthy Ageing, University College London, London, United Kingdom; ^2^Faculty of Natural Sciences, University of Kent, Canterbury, United Kingdom; ^3^Institute of Biodiversity, Animal Health and Comparative Medicine, University of Glasgow, Glasgow, United Kingdom

**Keywords:** RNA polymerase III, ageing, mTOR, TORC1, MAF1

## Abstract

Transcription in eukaryotic cells is performed by three RNA polymerases. RNA polymerase I synthesises most rRNAs, whilst RNA polymerase II transcribes all mRNAs and many non-coding RNAs. The largest of the three polymerases is RNA polymerase III (Pol III) which transcribes a variety of short non-coding RNAs including tRNAs and the 5S rRNA, in addition to other small RNAs such as snRNAs, snoRNAs, SINEs, 7SL RNA, Y RNA, and U6 spilceosomal RNA. Pol III-mediated transcription is highly dynamic and regulated in response to changes in cell growth, cell proliferation and stress. Pol III-generated transcripts are involved in a wide variety of cellular processes, including translation, genome and transcriptome regulation and RNA processing, with Pol III dys-regulation implicated in diseases including leukodystrophy, Alzheimer’s, Fragile X-syndrome and various cancers. More recently, Pol III was identified as an evolutionarily conserved determinant of organismal lifespan acting downstream of mTORC1. Pol III inhibition extends lifespan in yeast, worms and flies, and in worms and flies acts from the intestine and intestinal stem cells respectively to achieve this. Intriguingly, Pol III activation achieved through impairment of its master repressor, Maf1, has also been shown to promote longevity in model organisms, including mice. In this review we introduce the Pol III transcription apparatus and review the current understanding of RNA Pol III’s role in ageing and lifespan in different model organisms. We then discuss the potential of Pol III as a therapeutic target to improve age-related health in humans.

## Introduction

The transcription of the eukaryotic nuclear genome is performed by three, evolutionarily conserved, multi-subunit RNA polymerases (Pols) that each transcribe a distinct set of genes. A large proportion of the nuclear genome is transcribed by Pol II to generate both coding and non-coding RNAs. In contrast, Pol I only transcribes a single gene, albeit present in multiple copies within the genome, to produce the precursor to most rRNAs. While Pol I and III transcribe fewer genes, they generate some of the most abundant cellular RNAs accounting for much of the cellular transcriptional activity (White, 2008; Vannini and Cramer, 2012; Arimbasseri and Maraia, 2016).

With 17 subunits, Pol III is the largest of the three RNA polymerases (Vannini and Cramer, 2012). It transcribes several abundant, non-coding RNAs (up to a few hundred bases in length), some of which are involved in translation, such as the 5S rRNA and tRNAs, some are involved in RNA processing, such as a number of sn or snoRNAs, whereas others play regulatory roles, such as 7SK RNA. Indeed, the list of Pol III-transcribed genes has been expanding in recent times (Acker et al., 2013; Turowski and Tollervey, 2016). Pol III function has also extended beyond the canonical role in transcription of the nuclear genome to now include responses to DNA viruses and homologous recombination-mediated repair of DNA double-strand breaks (Chiu et al., 2009; Liu et al., 2021). Pol III mediated transcription is involved in a wide range of biological processes including cell and organismal growth (Grewal et al., 2005, 2007; Halaschek-Wiener et al., 2005; Chiu et al., 2009; Marshall et al., 2012; Rideout et al., 2012), cell cycle (White et al., 1995), stemness and differentiation (Wong et al., 2011; Alla and Cairns, 2014; Van Bortle et al., 2017; Chen et al., 2018), development (Schmitt et al., 2014), regeneration (Yeganeh et al., 2019), and cellular responses to stress (Upadhya et al., 2002; Gouge et al., 2015). As a result, Pol III subunits have been implicated in a wide variety of disease states, reviewed by (Yeganeh and Hernandez, 2020).

## Pol III Transcriptional Machinery

Recruitment of Pol III to its target genes is facilitated by a number of dedicated, basal transcription factors (TFs), where the exact combination of TFs involved is dictated by the particular promoter present (reviewed in Schramm and Hernandez (2002). Three types of promoter (Type I-III) recruit Pol III, with all three requiring the binding of TFIIIB, a 3-subunit TF containing the TATA box-binding protein (TBP). Type I and Type II promoters are gene-internal, while type III reside in the 5′ flanking region. Type II is present in tRNA genes and additionally requires TFIIIC, a 6-subunit TF that binds intragenic promoter elements. Type I promoter is responsible for 5S rRNA gene transcription and employs a further factor, TFIIIA, to direct TFIIIC binding. Type III are distinct from Type I and II promoters as they do not require TFIIIC for Pol III mediated transcription but employ SNAPc, a TF also associated with Pol II transcription (Schramm and Hernandez, 2002). This general set of promoter types and TFs is essentially conserved across wide evolutionary distances but with some phyla-specific differences (Schramm and Hernandez, 2002; Teichmann et al., 2010). Additional TFs regulating Pol III activity include Myc, a transcriptional activator that can act on all three Pols (Gomez-Roman et al., 2003; Campbell and White, 2014), as well as the protein Maf1, a highly conserved repressor of Pol III activity (Upadhya et al., 2002; Vorländer et al., 2020). Due to the critical functions of RNA Pol III in growth and differentiation, it is perhaps not altogether surprising that the signalling pathways influencing these cellular processes can also interact and regulate Pol III activity (Willis and Moir, 2018). Probably the most studied of these is the highly conserved mTOR pathway (Wei et al., 2009; Liu and Sabatini, 2020), which we will discuss in the following section.

## Ageing and the mTOR Pathway

The increased proportion of older people within our societies has stimulated the study of the biology of ageing with the hope that thorough understanding the mechanisms of ageing we will come closer to identifying targets for intervention to help prevent or ameliorate diseases of ageing (Partridge et al., 2018). Indeed, several decades of research have shown that ageing itself is highly plastic and can be modulated through genetic, dietary and pharmacological means. For example, reduction in the activity of the Target of Rapamycin kinase Complex 1 (TORC1) can promote longevity and healthspan in a number of animal species (Erdogan et al., 2016; Liu and Sabatini, 2020). The central component of the complex is mTOR, a 289 kDa Ser/Thr kinase belonging to the PI3K-related protein kinase (PIKK) family (Liu and Sabatini, 2020). TORC1 is activated in the presence of nutrients and growth factors, essentially acting to promote anabolic pathways while supressing catabolism. Given that protein synthesis is one of the most highly energy-intensive anabolic processes required for growth (Buttgereit and Brand, 1995), TORC1 tightly regulates the provision of protein synthetic machinery, including regulation of Pol III activity. Indeed, TORC1 has also been shown to localise to the promoters of a number of rRNA and tRNA genes and control their transcription (Li et al., 2006). It is this link between TORC1 and Pol III that initially prompted us to investigate the role of Pol III in organismal ageing.

## RNA Polymerase III Acts to Promote Organismal Ageing

It is possible to reduce the activity of RNA Polymerase III in model organisms by downregulating individual subunits of this complex, through either a partial or restricted loss of function, thereby avoiding organismal lethality that follows complete loss. We found that in the nematode worm *Caenorhabditis elegans*, RNAi targetted to the gene *rpc-1*, which encodes the largest of the 17 Pol III subunits (orthologue of yeast RPC160), significantly extended organismal lifespan (Filer et al., 2017). This was also true in the fruit fly *Drosophila melanogaster*, where RNAi of dC160 (the fly orthologue of RPC160) extended lifespan, as did a heterozygous mutant of another Pol III subunit dC53 (Filer et al., 2017). Indeed, inducible loss of RPC160 in yeast also extended chronological lifespan, thus demonstrating extensive evolutionary conservation (Filer et al., 2017).

Since the longevity of an organism can be determined by a specific organ, we tested whether this was also the case for Pol III (Filer et al., 2017). As the intestine has previously been shown to be important for modulating longevity in both worms and flies, we focussed our attention on this tissue (Libina et al., 2003; Piper et al., 2008). We showed that inhibiting Pol III activity in the adult worm or fly gut using tissue-specific RNAi was sufficient to extend lifespan; and more specifically in flies, longevity was also achieved by Pol III inhibition exclusively within intestinal stem cells (ISCs) (Filer et al., 2017). In contrast, downregulation of Pol III in the neurons and fat body of flies had little or no effect on lifespan (Filer et al., 2017).

With advancing age, the function of many organ systems and tissues deteriorates, contributing to physiological decline, multimorbidity and ultimately death (Rera et al., 2012; Ezcurra et al., 2018; Funk et al., 2020). For example, in worms and flies the intestinal luminal wall begins to break down and becomes more porous with advancing age, with similar changes in intestinal permeability and barrier function reported in mammals, including humans (Funk et al., 2020). We found that the long life caused by Pol III knockdown was associated with amelioration of age-related gut pathology and its ensuing functional decline (Filer et al., 2017). Critically this suggests that Pol III reduction is both a target for longevity and for age-related health, and together with the intestinal longevity data intestinal longevity points to tissue-specific functions of the Pol III complex.

## RNA Polymerase III as a Downstream Effector of TORC1

Importantly, we have also identified Pol III as a downstream effector of TORC1 (Filer et al., 2017). Rapamycin is a macrolide compound which inhibits activity of the TOR kinase and rapamycin treatment extends lifespan in a range of organisms (Bjedov et al., 2010; Arriola Apelo and Lamming, 2016), with rapamycin treatment in flies decreasing pre-tRNAs in both whole flies and within the intestine (Filer et al., 2017). Indeed, we found that whilst both rapamycin and intestine- or ISC-specific Pol III knockdown extended lifespan to a similar extent, these treatments were not additive (Filer et al., 2017). These findings led us to conclude that limiting Pol III activity in the fly adult gut achieves the full longevity benefit of systemic TORC1 inhibition and that Pol III is a key output of this highly conserved nutrient signalling pathway that determines lifespan (Filer et al., 2017).

**FIGURE 1:**
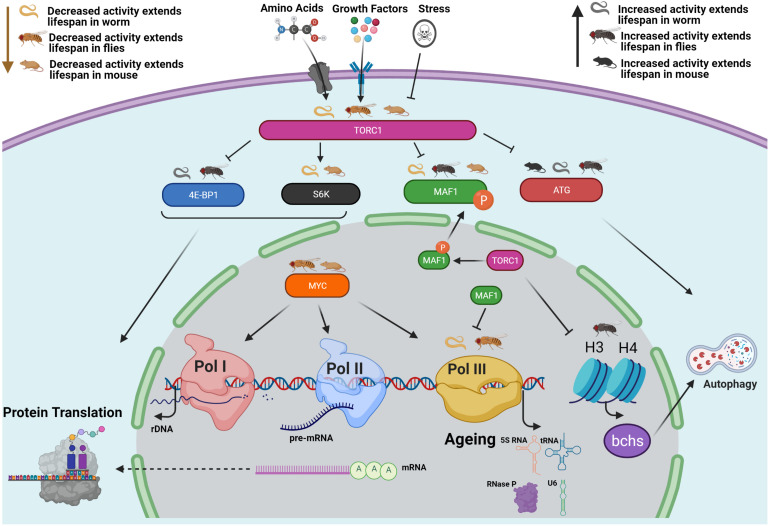
Conserved relationships between TORC1, RNA Polymerase III and lifespan. The growth-promoting, anabolic functions mediated by Pol III activation represent at least one mechanism downstream of TORC1 that shortens adult health and survival (Filer et al., 2017). The “Odd Pols” Pol I and Pol III have also been implicated in disease states (Sharifi and Bierhoff, 2018; Zhang et al., 2018). TORC1 also controls the translational regulators S6 Kinase and 4E-BP1 as well as the autophagy regulators ATG1 and ULK1 (Liu and Sabatini, 2020), and the H3 H4 Axis (Lu et al., 2021). NB Histones H3/H4 regulate expression of an autophagy cargo adaptor Bchs (WDFY3 in mammals). Pol III is also regulated by MYC (as are Pol I and Pol II) which has also been implicated in the TORC1 pathway and in ageing (Gomez-Roman et al., 2003; Campbell and White, 2014; Hofmann et al., 2015; Zhao et al., 2019). This figure focuses on work in the worm, fly and mouse. Studies in these three model systems demonstrate that all of these TORC1 outputs can affect lifespan, as summarised here. As the structure and biochemical function of Pol III is well studied, it is possible to design inhibitors that may provide therapeutic effects (Abascal-Palacios et al., 2018; Girbig et al., 2021).

Our work shows that the growth-promoting, anabolic functions mediated by Pol III activation are at least one mechanism downstream of TORC1 that results in TORC1’s activity curtailing adult health and survival (Filer et al., 2017; Figure 1). Other known effectors of TORC1 include the translational regulators S6 Kinase and 4E-BP1, in addition to the autophagy regulators ATG1 and ULK1 (Liu and Sabatini, 2020). Inhibition of TORC1 with rapamycin also increases levels of Histone H3 and H4 to induce autophagy (Lu et al., 2021). Studies in multiple model systems demonstrate that these TORC1 outputs can all affect lifespan (Hansen et al., 2007; Pan et al., 2007; Selman et al., 2009; Zid et al., 2009; Carvalho et al., 2017; Fernández et al., 2018; Stead et al., 2019; Lu et al., 2021). TORC1 is part of a complex signalling network, the interactions of which rarely work in isolation; the interaction between Pol III and each of TORC1’s outputs remains to be defined (Figure 1).

## Myc and Longevity

All three polymerases are regulated by the transcription factor Myc, which has also been implicated both in the TORC1 pathway and in ageing (Gomez-Roman et al., 2003; Campbell and White, 2014; Hofmann et al., 2015; Zhao et al., 2019). Activation of TORC1 by amino acids requires the high affinity glutamine transporter SLC1A5, which itself is transcribed by Myc (Zhao et al., 2019). Reduced expression of Myc in Myc heterozygous loss of function mice also leads to reduced TORC1 signalling, increases mouse lifespan, and improves a number of age-related health conditions including osteoporosis, cardiac fibrosis, immunosenescence and several parameters associated with activity and lipid metabolism (Hofmann et al., 2015). This link between Myc and longevity is conserved in the fly (Greer et al., 2013). Thus, reduced activity of Myc is suggested as an anti-ageing therapy target. Given that Myc is able to activate Pol III transcription, it will be worth considering the interactions between Myc, Pol III and ageing in the future.

## Maf1 and Longevity

Previous research has shown that mTORC1 directly phosphorylates Maf1, a key regulator of Poll III function (Michels et al., 2010). Unphosphorylated Maf1 binds to the polymerase and a subunit of TFIIIB preventing recruitment of Pol III to promoters (Willis and Moir, 2018). Upon phosphorylation by mTORC1, Maf1 is translocated from the nucleus to the cytoplasm thus relieving Pol III from Maf1 repression (Oficjalska-Pham et al., 2006). For this review we are primarily focussed on the role of Maf1 in regulating Pol III target genes, although it should be noted that Maf1 may also regulate Pol I and Pol II transcription in multiple species (Johnson et al., 2007; Khanna et al., 2014; Palian et al., 2014; Zhang et al., 2018).

A number of studies by the Willis group have investigated the role of Maf1 in metabolism and longevity in mice (Bonhoure et al., 2015, 2020; Willis et al., 2018). They found that global Maf1 KO mice are resistant to both obesity and fatty liver disease when fed a high fat diet (Bonhoure et al., 2015). Interestingly, female Maf1 KO mice maintained on a chow diet had a significantly extended lifespan relative to WT mice (Bonhoure et al., 2015). While some of the beneficial metabolic effects observed upon loss of Maf1 appear linked to energetically costly, futile RNA cycling due to Pol III activation (Willis et al., 2018), it is still possible that they may also be mediated in part by Pol II (see above).

In parallel, several groups have examined effects of Maf1 on lifespan in invertebrates and yeast, with the findings appearing somewhat inconsistent. The deletion of Maf1 in the budding yeast *S. cerevisiae* shortened lifespan under high calorie (2% glucose) and caloric restriction (CR) feeding and decreased resistance to both heat and hydrogen peroxide (Cai and Wei, 2015, 2016). Similarly, loss of Maf1 in *S. pombe* resulted in shortened chronological lifespan under low glucose conditions (Shetty et al., 2020). In contrast, the loss of the Maf1 orthologue MAFR-1 in *C. elegans* through RNAi knockdown extended lifespan under normal and CR feeding, and increased stress resistance (Cai and Wei, 2016). More recently, the Curran lab employed a CRISPR- based approach to generate a *mafr-1* null mutant and reported no difference in the lifespan relative to WT worms (Hammerquist and Curran, 2020), despite showing some phenotypic overlap (e.g., increased expression of Pol III transcripts and elevated intracellular lipids) with the *mafr-1 RNAi* knockdown worms studied by Cai and colleagues (Cai and Wei, 2015, 2016). In *Drosophila*, *dMaf1* inhibition during development in either the whole organism or the fat body increased larval growth rates and body size, and enhanced protein synthesis rates through increased insulin signalling (Rideout et al., 2012), while over-expression in the adult gut moderately promoted longevity (Filer et al., 2017). Taken together, these complex observations made whilst studying Maf1 demonstrate a role for Maf1 in health and longevity which could be mediated by the role of Maf1 in controlling Pol III. In particular, they raise an intriguing possibility that both inhibition and activation of Pol III can promote health and longevity, dependent on context such as diet, tissue or organ where the intervention is implemented or even the species being examined. However, an alternative explanation is that the longevity of Maf1 loss of function is mediated by its action on Pol I or Pol II.

## Mechanisms of Longevity Incurred by Pol III Inhibition

As with all interventions that affect longevity it is critically important to understand their mechanisms of action and whether these mechanisms are conserved. We will now discuss several putative mechanisms that may underlie the longevity phenotypes associated with inhibition of Pol III, although it is important to note that these putative mechanisms are not mutually exclusive.

Reduced protein translation is a well-established longevity mechanism that may well underlie the longevity of Pol III inhibition. Pol III is responsible for driving the transcription of multiple small RNAs many of which play critical (but distinct) roles in driving protein translation (Ikegami and Lieb, 2013; Gruber, 2014; Filer et al., 2017; Pradhan et al., 2017; Sin et al., 2017). Indeed, work on Pol III inhibition and lifespan focuses specifically on its knockdown in adulthood, as blocking its ability to promote translation during development is lethal. For example, RNAi of *rpc-1* in worms from embryonic stages causes embryonic lethality, while loss-of-function mutation in dC53 is homozygous lethal (Filer et al., 2017). Similarly, mice homozygous for certain Pol III mutations are embryonic lethal at day E3.5 or are postnatally stunted dying between P16 and P25 (Choquet et al., 2019; Wang et al., 2020). Together, these developmental defects demonstrate the importance of Pol III’s provision of components of translation machinery for basic physiological processes and strongly suggest that a careful balance of translational capacity is required for optimal function, health and longevity. Related to this, non-coding RNAs such as tRNAs and rRNAs can also be the source of tRNA and rRNA fragments (tRFs and rRFs). These RNA fragments play a role in translation and are implicated in several age-related diseases (Guzzi and Bellodi, 2020) and the ageing process. It is possible that RNA Pol III could influence their biogenesis either directly or indirectly.

Whether it is a reduction in global translation or a reduction in translation of specific RNA species/families that contribute to longevity is yet to be determined. The balance between protein synthesis rates and quality control mechanisms to maintain proteostasis is also critical during ageing (Riera et al., 2016; Sands et al., 2017; Francisco et al., 2020). Given that the longevity phenotype of Pol III gut-specific inhibition in *Drosophila* is associated with enhanced resistance to proteotoxic challenge (Filer et al., 2017), we suggest that investigating these processes more fully in the context of Pol III across different organisms is both timely and relevant.

*G*enomic instability and the accretion of genetic damage to both genomic and mitochondrial DNA has been proposed as one of the central hallmarks of ageing (López-Otín et al., 2013). Protection against this damage in multicellular organisms is complex and takes many forms, evidence of which can be found in systems where Pol III activity is altered. In *S. Pombe*, when Pol III is activated by the loss of *maf1*, increased DNA damage of various tRNA genes was observed, but only significantly so under CR conditions but not high-calorie conditions (Shetty et al., 2020). Similarly, the Pol III-Maf1 axis has been shown to play a role in protecting tRNA genes from DNA damage in fission yeast, and can also respond to the DNA damage response (Hammerquist et al., 2021; Noguchi et al., 2021). Pol III has also been shown to produce transitory R-loops in double strand breaks required for homologous recombination repair (Liu et al., 2021). This method of double strand break repair is known to be important for telomeres, thus Pol III may play a role in telomere biology (Marnef and Legube, 2021; Tan et al., 2021). Hence, it appears possible that reducing Pol III activity could potentially impact ageing though reduced levels of DNA damage (Callegari, 2016).

Finally, Pol III may also play an important, yet somewhat under-appreciated role, in cellular metabolic processes. For example, in yeast reduced Pol III activity is associated with *de novo* amino acid synthesis, a decreased abundance of glycolytic enzymes and reduced glycolytic flux. In contrast, in the same study enhanced glycolytic flux was observed in Maf1-deficient yeast cells (Cieśla et al., 2007; Szatkowska et al., 2019), with evidence of greater hepatic glycolytic flux in fed Maf1 KO mice (Willis et al., 2018). Thus, taken together there are several candidate mechanisms via which Pol III inhibition may act to increase lifespan and healthspan. Most are well established longevity processes; deciphering exactly how they act or combine to orchestrate the effects of Pol III will be intriguing.

## Summary

The evolutionary conservation of Pol III affirms its potential as an exciting, novel therapeutic target for ageing and age-related health. This conservation across organisms that span wide evolutionary distances lends itself to an integrated-organismal approach to its study. The underlying mechanisms may be complex due to cross-talk between systems controlling gene expression (Policarpi et al., 2017; Gerber et al., 2020). However, the structure and biochemical function of Pol III is well studied, providing a basis for design of polymerase inhibitors (Abascal-Palacios et al., 2018; Girbig et al., 2021). Through a better understanding of Pol III regulation, its transcriptional outputs, the relevance of its tissue specificity and how mechanistically it acts to modulate physiological function and health across the life-course in distinct model systems (e.g., worms, flies, mice), we believe it will be possible to exploit the specific advantages of each system in order to maximise knowledge output on this important lifespan determinant.

## Author Contributions

YK, YM, and GB contributed to the writing of the manuscript. JT, NA, and CS supervised and edited the manuscript. All the authors contributed to the article and approved the submitted version.

## Conflict of Interest

The authors declare that the research was conducted in the absence of any commercial or financial relationships that could be construed as a potential conflict of interest.
