# Corrigendum: RNA Polymerase III, Ageing and Longevity

**DOI:** 10.3389/fgene.2021.758135

**Published:** 2021-09-02

**Authors:** Yavuz Kulaberoglu, Yasir Malik, Gillian Borland, Colin Selman, Nazif Alic, Jennifer M. A. Tullet

**Affiliations:** ^1^Department of Genetics Evolution and Environment, Institute of Healthy Ageing, University College London, London, United Kingdom; ^2^Faculty of Natural Sciences, University of Kent, Canterbury, United Kingdom; ^3^Institute of Biodiversity, Animal Health and Comparative Medicine, University of Glasgow, Glasgow, United Kingdom

**Keywords:** RNA polymerase III, ageing, mTOR, TORC1, MAF1

In the published article, there was an error regarding the affiliations for Nazif Alic. Nazif Alic should only have affiliation 1.

The authors apologize for this error and state that this does not change the scientific conclusions of the article in any way. The original article has been updated.

## Publisher's Note

All claims expressed in this article are solely those of the authors and do not necessarily represent those of their affiliated organizations, or those of the publisher, the editors and the reviewers. Any product that may be evaluated in this article, or claim that may be made by its manufacturer, is not guaranteed or endorsed by the publisher.

